# Non-Linear Association between Obstructive Sleep Apnea Risk and Lipid Profile: Data from the 2015–2018 National Health and Nutrition Examination Survey

**DOI:** 10.31083/j.rcm2505175

**Published:** 2024-05-17

**Authors:** Gaoyuan Ge, Dan Bo, Fengxiang Zhang, Di Yang

**Affiliations:** ^1^Department of Cardiology, The First Affiliated Hospital of Nanjing Medical University, 210029 Nanjing, Jiangsu, China; ^2^Department of Cardiology, The Affiliated Hospital of Yangzhou University, 225000 Yangzhou, Jiangsu, China

**Keywords:** obstructive sleep apnea, dyslipidemia, physical activity, cardiovascular mortality

## Abstract

**Background:**

The relationship between the multivariable apnea prediction (MAP) index and lipid levels was examined using a cross-sectional and retrospective study of National Health and Nutrition Examination Surveys (2015–2018). A total of 3195 participants with MAP scores were included in the analysis.

**Methods:**

The MAP index, an algorithm leveraging sleep apnea symptom frequency, body mass index (BMI), age, and sex, estimates the risk of obstructive sleep apnea (OSA). We investigated the associations between the MAP index and lipid profiles—specifically, high-density lipoprotein cholesterol (HDL-C), total cholesterol (TC), low-density lipoprotein cholesterol (LDL-C), and triglycerides (TG) —using weighted linear regression and restricted cubic splines (RCS) analysis. Additionally, mediation analysis was conducted to explore the potential mediating role of physical activity on the link between OSA risk, hyperlipidemia, and cardiovascular mortality.

**Results:**

A non-linear relationship was observed between OSA severity and lipid profiles, including elevated levels of TC, increased LDL-C, higher TG, and decreased HDL-C (All *p* for non-linearity <0.05). The findings remained consistent across the stratified sensitivity analyses. Furthermore, physical activity served as a mediator in the association between the MAP index and both hyperlipidemia and cardiovascular mortality, accounting for 16.6% and 16.7% of the indirect effects, respectively.

**Conclusions:**

Participants at high risk for OSA demonstrated an increased prevalence of dyslipidemia. Additionally, engagement in physical activity was shown to have beneficial effects on lipid metabolism.

## 1. Introduction

Obstructive sleep apnea (OSA) and dyslipidemia are well-established risk factors for cardiovascular disease (CVD) [1]. Intermittent hypoxia (IH) and sleep fragmentation are the most crucial factors in OSA pathogenesis, and result in overdrive of the sympathetic system, oxidative stress, and systemic inflammation [2]. These disturbances lead to metabolic imbalances characterized by decreased high-density lipoprotein cholesterol (HDL-C) as well as increased total cholesterol (TC), low-density lipoprotein cholesterol (LDL-C), and triglycerides (TG) [3]. It has been established that obesity exacerbates the impact of dyslipidemia, which is intensified by IH [4], and significantly impacts sleep disturbances [5, 6]. Furthermore, the male sex and higher apnea–hypopnea index scores were associated with reduced hypo-HDL-C levels and a hyper-TC/HDL-C ratio [7]. Although the prevalence of both OSA and dyslipidemia rise with age [4, 8, 9], there is limited research on the synergistic effects of these risk factors in exacerbating hyperlipidemia.

The multivariable apnea prediction (MAP) index is an algorithm that assesses OSA risk. It incorporates sleep apnea symptom frequency, body mass index (BMI), age, and sex, making it a valuable screening tool [10]. Utilizing this index allows for precise risk stratification among participants suspected of having OSA.

While numerous studies acknowledge the link between OSA and dyslipidemia [5, 11], these studies often fail to control for confounding factors such as diet, regular exercise, or lipid-lowering medications, which may introduce bias. To mitigate these limitations, our study utilizes a representative sample from the National Health and Nutrition Examination Survey (NHANES) 2015–2018. Here, we examine the relationship between OSA and blood lipid levels, specifically evaluating the impact of physical activity on this association.

## 2. Methods

### Data and Study Population

This study utilized data obtained from the NHANES database, an annual survey conducted by the National Center for Health Statistics (NCHS), a division of the Centers for Disease Control and Prevention (CDC). The survey aims to assess the health nutritional status and health behaviors of the unstructured population in the United States. A complex, multi-stage probability sampling design is used in the NHANES survey to obtain representative data. All NHANES protocols were implemented in accordance with the United States Department of Health and Human Services (HHS) human research subject protection policy and are reviewed and standardized annually by the NCHS Research Ethics Review Committee. All subjects participating in the survey have signed informed consent. All data used in this study were published free of charge by NHANES and required no additional authorization or ethical review.

This study utilized data from two NHANES survey cycles (2015–2016 and 2017–2018), focusing on participants with available serum lipid measurements. We excluded 1301 participants due to incomplete demographic, clinical, and lifestyle factor data. The final analysis included 3195 participants, although survival data were missing for eight participants. **Supplementary Fig. 1** shows details of the overall study design, sampling, and exclusion criteria.

## 3. Measures

### 3.1 The MAP Index Score

The MAP index is a screening tool for OSA. Two questions on frequency of symptoms including “snoring” and “snort or stop breathing” were recoded to approximate the original MAP questions. Item responses are rated 0 to 4: 4 = always (5–7 times per week); 3 = frequently (3–4 times per week); 2 = sometimes (1–2 times per week); 1 = rarely (less than once a week); 0 = never; and do not know (missing data point). An apnea score was calculated, as in the original multivariable apnea prediction, using the available responses along with BMI, age, and sex into the adapted MAP algorithm. The original multivariable apnea prediction had good test-retest reliability following a 2 week period (correlation coefficient, 0.92). It’s effectiveness in identifying OSA based on an original multivariable apnea prediction score of 0.50 or greater (Apnea Hyperpnea Index ≥10) was characterized by high sensitivity (88%; 95% CI, 84%–92%) but lower specificity (55%; 95% CI, 48%–62%) among sleep clinic patients [10].

### 3.2 Lipid Profile and Hyperlipidemia

Participants were asked to fast overnight before the blood draw. Serum TC (mg/dL) and TG (mg/dL) levels were determined by the enzymatic methods using the Hitachi Automatic Analyzer 7600-210 (Hitachi, Tokyo, Japan). The homogeneous enzymatic colorimetric method was used to assess the serum HDL-cholesterol level (mg/dL). Adult Treatment Panel III (ATP 3) of the National Cholesterol Education Program (NCEP) classified hyperlipidemia [12] as TC 200 mg/dL, TG 150 mg/dL, HDL-C 40 mg/dL in males and 50 mg/dL in females, or LDL-C 130 mg/dL. Alternately, participants who reported using cholesterol-lowering drugs were also classified as having hyperlipidemia.

### 3.3 Self-Reported Physical Activity

Obtained from the Global Physical Activity Questionnaire, the questionnaire includes questions related to different domains of physical activity such as leisure time moderate-to-vigorous physical activity (MVPA). Physical activity was divided into two categories (no/yes), based on the frequencies of time spent in an activity. “No” describes physical activity equal to 0 min/week. “Yes” included physical activity greater than 0 min/week.

### 3.4 Cardiovascular Mortality

Cardiovascular mortality was defined according to the International Classification of Diseases (ICD-10) criteria. Underlying cause of death with ICD-10 codes of I00–I09, I11, I13, I20–I51, and I60–I69 were defined as death from cardiovascular disease.

### 3.5 Comorbid Conditions

Household questionnaires were conducted via home interview, and were used to identify participants with a history of arteriosclerotic cardiovascular disease (ASCVD). Hypertension was identified as mean systolic blood pressure ≥130 mmHg or diastolic blood pressure ≥80 mmHg, or if the participant was currently taking anti-hypertensive medication. Diabetes was recognized in participants having either diagnosed diabetes, fasting glucose ≥7.0 mmol/L, hemoglobin A1c ≥48 mmol/mol, and/or treatment with anti-diabetic medication.

### 3.6 Covariates

Demographic information and medical history were assessed using self-reported questionnaires. We investigated age, sex, ethnicity (white, black and other race), education level (less than high school, high school or GED, and above high school), and family poverty income ratio (PIR) as sociodemographic factors. Height, weight, and waist circumference were measured by trained clinicians using standardized examination methods and calibrated equipment. Body mass index was calculated from the measured height and weight.

Cigarette smoking status was modeled as a three-level variable: current smokers (smoked >100 cigarettes in their lifetime and reported smoking in the past 30 days), former smokers (smoked >100 cigarettes in their lifetime and did not report smoking in the past 30 days), and never smokers (never smoked 100 or more cigarettes). Alcohol consumption was similarly modeled as a three-category variable as well: none (<12 drinks in lifetime) or mild drinkers (past year ≤1 drink per day on average for women or ≤2 drinks per day for men), moderate drinkers (≤3 drinks per day for women or ≤4 drinks per day for men in the past year), and heavy drinkers (>3 drinks per day for women or >4 drinks per day for men in the past year). Healthy eating index values were calculated according to the Healthy Eating Index (HEI)-2015 guidelines [13], with higher scores indicating a better quality of diet. Hypolipidemic medication (within the past 30 days) was defined as no (no medication was used), and yes (use of lipid-lowering medications).

### 3.7 Statistical Analysis

The NHANES 2005–2018 MEC exam data weights were used in all analyses to take stratification and clustering into account because of the complex sample design.

Categorical variables were summarized as percentages, while continuous variables were reported as mean ± standard deviation. To analyze differences between continuous data, the Kruskal-Wallis test or one-way analysis of variance (ANOVA) was used, while the chi-square test was used to analyze differences between categorical data. The MAP index scores were categorized based on quartiles (Q1: ≤25th percentile, Q2: >25 to 50th percentile, Q3: >50 to 75th percentile, Q4: >75th percentile) for analysis. To assess the relationship between OSA risk and lipid profiles, we conducted a weighted multivariate linear regression and restricted cubic splines (RCS) analysis. The Crude Model remained unadjusted, model 1 was adjusted for race, education, PIR, waist circumference and take anti-hyperlipidemic drug, and model 2 was adjusted for model 1 variables plus smoking, drinking status, physical activity, energy intake, and HEI. This methodology was similarly applied to assess the relationship between OSA risk and lipid levels, as stratified by physical activity. Interactions were evaluated using the *p*-values for the production terms between OSA risk and physical activity.

The mediation analyses were carried out using the R package ‘medflex’. Mediation analysis was performed to examine the potential mediating role of physical activity on the relationship between OSA risk and hyperlipidemia. To investigate the potential mediating effect of physical activity on the association between MAP (exposure) and hyperlipidemia/cardiovascular mortality (outcome), we used multivariable regression analysis. All models were conducted adjusted for race, education, smoking, and taking anti-hyperlipidemic drug.

### 3.8 Patient and Public Involvement

Patients or the public were not involved in the design, conduct, reporting or dissemination plans of our research.

## 4. Results

### 4.1 Baseline Characteristics

The demographic and lifestyle characteristics across quartiles of the MAP score are presented in Table 1. The samples with a higher OSA risk exhibited higher incidences of previous smoking, heavy drinking, increased daily energy intake, reduced physical activity, and more frequent use of anti-hyperlipidemic drugs. However, no significant differences were observed in the Healthy Eating Index HEI scores.

**Table 1.** **Demographic and lifestyle characteristics of participants by quartiles of MAP index score**.

		OSAS MAP score				
		(N = 3195)				
Variable		Q1	Q2	Q3	Q4	*p* value
		(n = 797)	(n = 799)	(n = 801)	(n = 798)	
Age (years)		38.04 (0.88)	45.92 (0.86)	51.28 (0.96)	56.86 (0.63)	<0.01
Gender (%)						<0.01
	Male	21.60 (2.61)	40.86 (2.88)	60.82 (2.22)	81.48 (2.67)	
	Female	78.40 (2.61)	59.14 (2.88)	39.18 (2.22)	18.52 (2.67)	
Race (%)						0.02
	White	64.57 (3.07)	60.58 (2.39)	68.22 (3.01)	69.59 (3.45)	
	Black	10.19 (1.46)	12.14 (1.82)	8.83 (1.54)	10.07 (1.79)	
	Other	25.24 (2.54)	27.28 (1.82)	22.95 (2.19)	20.34 (2.65)	
Education (%)						0.01
	Less than high school	7.63 (1.30)	11.10 (1.15)	12.47 (1.65)	13.13 (1.77)	
	High school or GED	21.84 (2.07)	27.96 (2.82)	21.69 (2.71)	27.10 (2.62)	
	Above high school	70.53 (2.62)	60.93 (2.92)	65.84 (2.82)	59.77 (3.09)	
Poverty		2.99 (0.08)	2.98 (0.08)	3.11 (0.10)	3.24 (0.10)	0.07
Smoke (%)						<0.01
	Never	65.24 (2.44)	58.57 (2.82)	51.83 (2.50)	48.62 (2.30)	
	Former	17.77 (2.29)	23.50 (2.74)	28.96 (2.41)	36.66 (2.83)	
	Now	17.00 (1.68)	17.92 (2.28)	19.21 (1.91)	14.72 (1.68)	
Alcohol user (%)						<0.01
	None or mild	50.07 (2.77)	59.10 (2.92)	61.82 (2.98)	68.89 (3.01)	
	Moderate	26.75 (2.57)	21.79 (2.07)	18.20 (2.24)	12.16 (1.83)	
	Heavy	23.18 (2.05)	19.10 (2.07)	19.98 (2.23)	18.94 (2.17)	
Physical activity (%)						<0.01
	Yes	66.96 (2.91)	56.31 (2.18)	52.93 (2.96)	49.02 (3.16)	
Energy (kcal/day)		1974.42 (46.36)	2089.57 (44.07)	2276.94 (50.25)	2401.48 (56.47)	<0.01
HEI score (%)						0.15
	Q1	21.59 (1.71)	27.21 (2.17)	26.70 (2.69)	24.94 (2.07)	
	Q2	27.17 (2.26)	21.51 (2.45)	25.77 (2.35)	28.26 (2.27)	
	Q3	23.35 (1.95)	28.17 (2.06)	25.56 (2.34)	25.74 (2.17)	
	Q4	27.89 (2.93)	23.11 (2.63)	21.97 (2.07)	21.05 (2.13)	
Anti-hyperlipidemic drug (%)						<0.01
	Yes	4.66 (1.17)	15.94 (1.71)	24.90 (1.94)	37.33 (2.65)	

HEI, Healthy Eating Index; MAP, multivariable apnea prediction; OSAS, obstructive sleep apnea syndrome; GED, general educational development.

In analyzing the relationship between the MAP index scores and various health indicators, a clear gradient is observed across the quartiles. Specifically, waist circumference, TG, TC and LDL-C were higher in the samples with higher in participants with elevated MAP scores, highlighting a direct correlation with increased OSA risk. Notably, participants in the highest MAP score quartile not only demonstrated significantly larger waist circumferences—averaging 116.71 cm compared to 84.60 cm in the lowest quartile—but also exhibited heightened levels of TG and LDL-C, with TG levels rising from 0.89 mmol/L in the first quartile to 1.49 mmol/L in the fourth. Furthermore, this group showed a marked predisposition towards ASCVD, diabetes mellitus (DM), and hypertension. For instance, the prevalence of ASCVD surged from 1.17% in the first quartile to 15.21% in the fourth, diabetes prevalence increased from 4.48% to 31.77%, and hypertension rates escalated from 21.68% to 74.27%, underscoring the significant health impacts associated with higher OSA risk. These findings, detailed in Table 2, emphasize the critical link between OSA risk and adverse health outcomes, particularly in terms of cardiovascular and metabolic health.

**Table 2.** **Clinical and biochemical data of participants by quartiles of OSAS MAP score quantiles**.

		OSAS MAP score				
		(N = 3195)				
Variable		Q1	Q2	Q3	Q4	*p* value
		(n = 797)	(n = 799)	(n = 801)	(n = 798)	
BMI (Kg/$m^{2}$)		23.71 (0.21)	28.19 (0.24)	31.02 (0.37)	35.66 (0.42)	<0.01
Waist circumference (cm)		84.60 (0.54)	96.84 (0.55)	105.27 (0.93)	116.71 (0.81)	<0.01
TC (mmol/L)		4.78 (0.05)	4.97 (0.06)	4.96 (0.05)	4.83 (0.08)	0.01
HDL-C (mmol/L)		1.67 (0.03)	1.45 (0.02)	1.37 (0.02)	1.23 (0.02)	<0.01
LDL-C (mmol/L)		2.70 (0.04)	2.97 (0.04)	2.98 (0.03)	2.92 (0.05)	<0.01
TG (mmol/L)		0.89 (0.02)	1.20 (0.03)	1.33 (0.04)	1.49 (0.06)	<0.01
ASCVD (%)						<0.01
	Yes	1.17 (0.47)	6.92 (1.37)	7.60 (1.08)	15.21 (1.85)	
DM (%)						<0.01
	Yes	4.48 (0.70)	11.33 (1.45)	21.36 (1.86)	31.77 (2.45)	
Hypertension (%)						<0.01
	Yes	21.68 (1.63)	45.04 (2.26)	61.19 (2.68)	74.27 (2.25)	

BMI, body mass index; TC, total cholesterol; HDL-C, high-density lipoprotein cholesterol; LDL-C, low-density lipoprotein cholesterol; TG, triglyceride; ASCVD, arteriosclerotic cardiovascular disease; DM, diabetes mellitus; OSAS, obstructive sleep apnea syndrome; MAP, multivariable apnea prediction.

### 4.2 Association between MAP Index and Lipid Profile

The results of weighted regression models, revealing distinct trends in lipid levels across MAP score quartiles, are illustrated in **Supplementary Table 1**. Compared to the lowest quartiles (Q1), the levels of TC increased with higher MAP scores: an elevation of 0.28 in the second quartile (Q2: 95% confidence interval [CI]: 0.14–0.45, *p* < 0.01), 0.35 in the third quartile (Q3: 95% CI, 0.14–0.55, *p* < 0.01), and 0.31 in the fourth quartile (Q4: 95% CI, 0.08–0.55, *p* = 0.02), as observed in model 2 (*p* for trend = 0.02). Similarly, higher quartiles (Q3 and Q4) were more likely to have elevated LDL-C (Q3: β, 0.38, 95% CI, 0.21–0.54, Q4: β, 0.37, 95% CI, 0.18–0.57) and TG (Q3: β, 0.29, 95% CI, 0.17–0.41, Q4: β, 0.37, 95% CI, 0.21–0.53) in model 2. Additionally, a negative correlation was found between HDL-C and MAP scores (Q2: β, –0.13, 95% CI, –0.21– –0.05, Q3: β, –0.16, 95% CI, –0.24– –0.08, Q4: β, –0.23, 95% CI, –0.34– –0.12) in model 2. These findings underscore the nuanced interaction between MAP scores and lipid levels, hinting at a complex underlying mechanism.

**Fig. 1.**
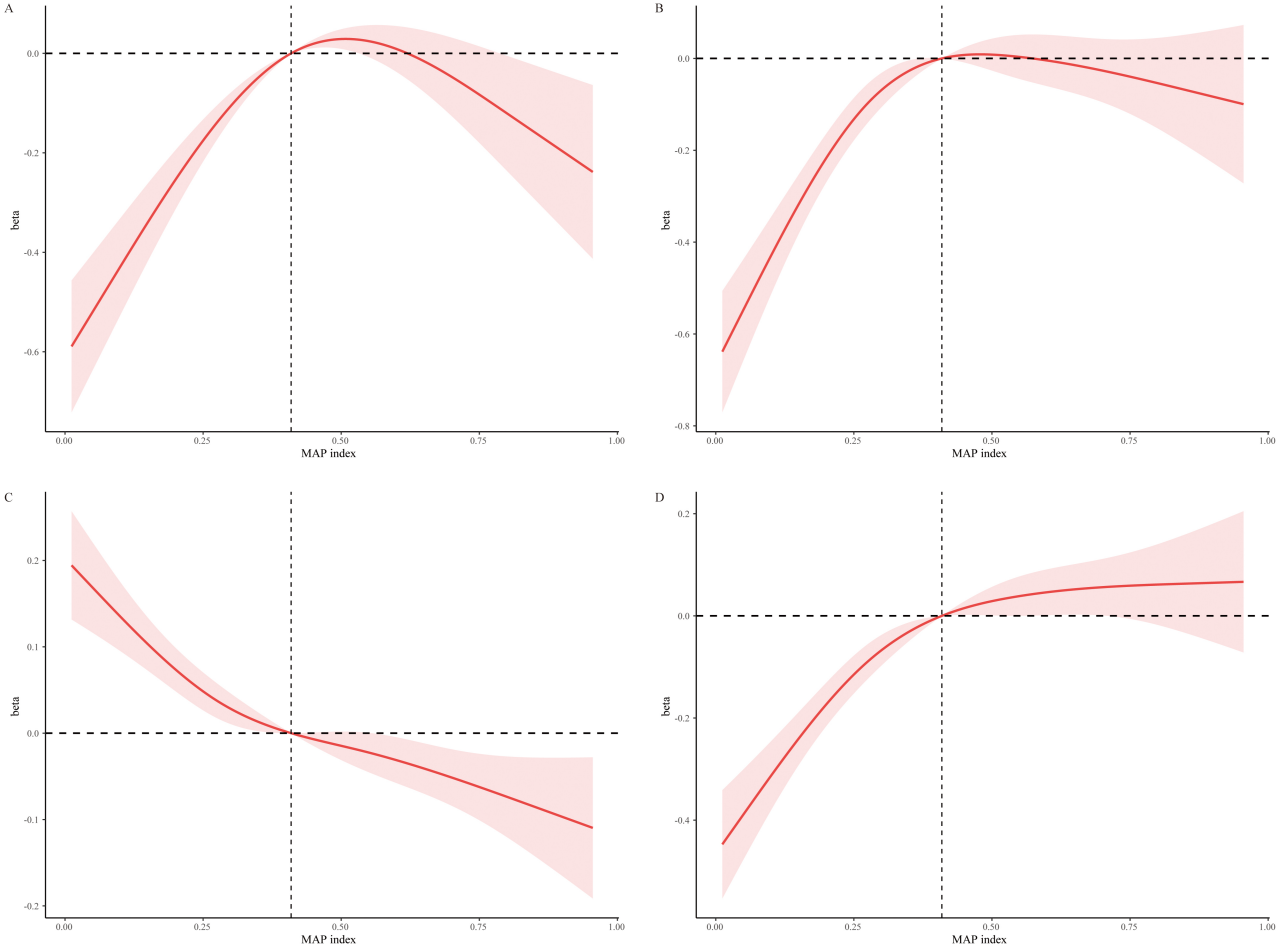
**Non-linear associations between lipid levels and MAP scores via restricted cubic spline regression models**. This figure illustrates the complex, non-linear relationships between MAP index scores and lipid levels (TC, LDL-C, HDL-C, and TG) using restricted cubic spline regression models. Adjustments were made for demographic and lifestyle variables including race, education level, poverty status, waist circumference, use of anti-hyperlipidemic drugs, smoking status, alcohol consumption, physical activity, energy intake, and Healthy Eating Index score. Panels: (A) TC. (B) LDL-C. (C) HDL-C. (D) TG. MAP, multivariable apnea prediction; TC, total cholesterol; LDL-C, low-density lipoprotein cholesterol; HDL-C, high-density lipoprotein cholesterol; TG, triglyceride.

We explored the relationships further through smooth curve fittings to evaluate the associations within the fully adjusted model. This analysis revealed apparent non-linear relationships between MAP scores and the levels of TC, LDL-C, and TG, with all showing significant trends of non-linearity (*p* for non-linearity < 0.05) (Fig. 1A,B,D). In contrast, a non-linear decrease in HDL levels was observed as MAP scores increased (Fig. 1C). Additionally, RCS analysis, focusing on the impact of OSA on lipid levels, detected a MAP inflection score at 0.41, indicating a critical threshold beyond which the lipid levels significantly alter.

### 4.3 Subgroup Analysis according to Physical Activity

Analysis of MAP scores and lipid profiles revealed consistent non-linear trends that were similar across subgroups stratified by physical activity, or exercise. Notably, MAP’s turning points were at 0.36 in the exercise group and 0.45 in the non-exercise group (**Supplementary Fig. 2**). While the interactions between OSA risk and levels of TC (*p* for interaction = 0.05) or LDL-C (*p* for interaction = 0.06) levels did not reach statistical significance in the non-exercise group, the trend was absent in physically active participants (*p* for trend: 0.29 and 0.18, respectively). Moreover, exercise did not significantly influence the relationship between OSA risk and LDL-C or TG levels, as all interactions showed *p*-values greater than 0.05 (Table 3).

**Table 3.** **Effects of physical activity on the association the between OSAS MAP index score quantiles and serum lipid levels**.

	Physical activity	No (n = 1599)		Yes (n = 1596)		*p* for interaction
	MAP index	β (95% CI)	*p*	β (95% CI)	*p*	
TC	Q1	ref		ref		0.05
	Q2	0.38 (0.20, 0.56)	<0.01	0.26 (0.07, 0.46)	0.01	
	Q3	0.53 (0.28, 0.77)	<0.01	0.23 (–0.08, 0.53)	0.13	
	Q4	0.54 (0.23., 0.85)	<0.01	0.12 (–0.22, 0.47)	0.46	
	*p* for trend		<0.01		0.29	
LDL-C	Q1	ref		ref		
	Q2	0.36 (0.20, 0.52)	<0.01	0.32 (0.16, 0.47)	<0.01	0.06
	Q3	0.48 (0.30, 0.66)	<0.01	0.31 (0.06, 0.55)	0.02	
	Q4	0.56 (0.34, 0.78)	<0.01	0.2 (–0.10, 0.49)	0.17	
	*p* for trend		<0.01		0.18	
HDL-C	Q1	ref		ref		
	Q2	–0.11 (–0.27, 0.05)	0.16	–0.13 (–0.22, –0.05)	0.01	0.12
	Q3	–0.11 (–0.28, 0.06)	0.19	–0.18 (–0.28, –0.09)	<0.01	
	Q4	–0.25 (–0.46, –0.04)	0.02	–0.19 (–0.30, –0.08)	<0.01	
	*p* for trend		0.01		0.01	
TG	Q1	ref		ref		0.71
	Q2	0.29 (0.12, 0.45)	<0.01	0.18 (0.06, 0.29)	0.01	
	Q3	0.35 (0.19, 0.50)	<0.01	0.23 (0.04, 0.43)	0.02	
	Q4	0.50 (0.27, 0.73)	<0.01	0.24 (0.05, 0.44)	0.02	
	*p* for trend		<0.01		0.02	

This table presents a subgroup analysis exploring the impact of physical activity on the relationship between quartiles of the OSAS MAP index score and serum lipid concentrations. The analysis is stratified into subgroups based on the presence (Yes) or absence (No) of physical activity. The model accounts for potential confounders, including race, education level, poverty status, waist circumference, use of anti-hyperlipidemic drugs, smoking status, alcohol consumption, energy intake, and Healthy Eating Index score. β, effect sizes; OSAS, obstructive sleep apnea syndrome; MAP, multivariable apnea prediction; TC, total cholesterol; HDL-C, high-density lipoprotein cholesterol; LDL-C, low-density lipoprotein cholesterol; TG, triglyceride; CI, confidence interval.

### 4.4 Mediation Analysis of the Effects of Physical Activity on the Association between MAP Index and Hyperlipidemia & Cardiovascular Mortality

**Fig. 2.**
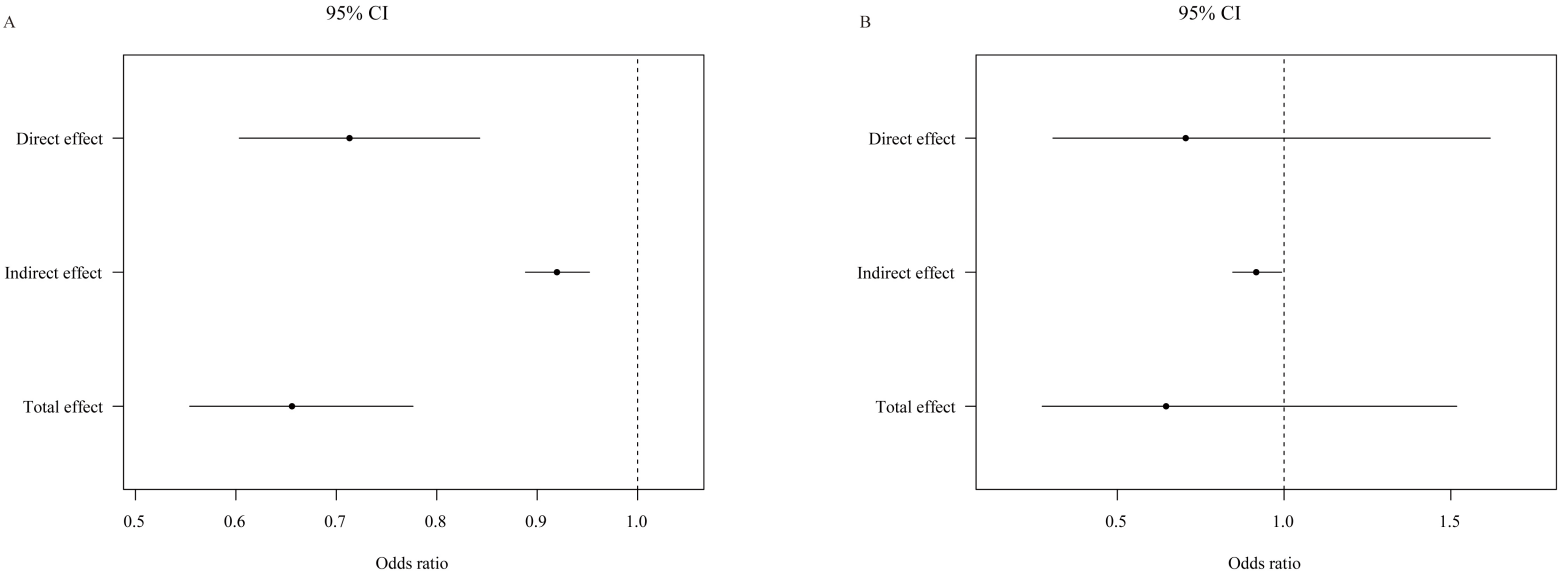
**Mediating role of physical activity in the association between MAP index, hyperlipidemia, and cardiovascular mortality**. This figure illustrates the mediating effects of physical activity on the association between MAP index scores and two critical health outcomes: hyperlipidemia and cardiovascular mortality. Panel A demonstrates the indirect impact of MAP scores on hyperlipidemia, mediated by physical activity. Panel B focuses on the effect of physical activity in the relationship between MAP scores and cardiovascular mortality risk. The analysis quantifies the extent to which physical activity can buffer the adverse effects of high MAP scores on these outcomes, providing insights into potential intervention points. The results, depicted in Panels A and B, emphasize the significance of physical activity in modulating the health risks associated with OSAS. MAP, multivariable apnea prediction; CI, confidence interval; OSAS, obstructive sleep apnea syndrome.

A statistically significant relationship was observed when physical activity was included as a mediator between MAP and hyperlipidemia (Fig. 2A). Notably, physical activity mediated 16.6% of MAP’s effect on hyperlipidemia (**Supplementary Table 2**). Although the direct impact of MAP on cardiovascular mortality did not reach statistical significance (odds ratio [OR]: 0.705, 95% CI: 0.307–1.618, *p* = 0.41), physical activity had a statistically significant impact on the relationship between MAP and cardiovascular mortality (OR: 0.916, 95% CI: 0.885–0.992, *p* = 0.03) (Fig. 2B).

## 5. Discussion

Our findings indicate a non-linear relationship between OSA risk and lipid profiles. The results demonstrated a strong association between OSA severity and increases to TC, LDL-C, and TG, alongside reductions in HDL-C. Notably, we found no direct significant interactions between physical activity and OAS on blood lipid levels. However, physical activity emerged as a significant factor mediating the effects of OSA on hyperlipidemia and cardiovascular mortality, highlighting its potential as a mitigating factor in OSA-related health risks.

The association between OSA and dyslipidemia is well-documented in the literature [3, 5, 14]. A crucial factor in OSA-associated hyperlipidemia is the impaired impaired clearance of circulating lipoproteins due to disrupted lipoprotein lipase (LpL) function [15]. Several mechanisms linked to OSA can influence LpL function, including IH [16], inflammation [17, 18], and hormonal changes [19]. Dyslipidemia, in turn, exacerbates endothelial dysfunction and consequently atherosclerosis, increasing the risk of cardiovascular mortality [15]. Therefore, it is imperative to assess blood lipid levels in OSA patients to more accurately assess and mitigate cardiovascular risk in clinical settings. Furthermore, after adjusting for potential confounders, we did not observe a positive correlation between MAP and TC or LDL-C at high MAP levels (above 0.41) levels. However, at low MAP levels (below 0.41), there was a trend of increasing association between MAP and TC or LDL-C, suggesting the possible influence of antihyperlipidemic drugs and lifestyle characteristics. Besides, among patients with elevated MAP (above 0.41) levels, there was a dose-response relationship between increased OSA risk and a slower decline in HDL-C, as well as a slower increase in TG.

In our study, we observed no significant interaction effect was observed between physical activity and MAP index on dyslipidemia, underscoring the robustness of the study findings. Moreover, the lipid profile trend in the physical exercise group remained relatively stable as the risk of OSA increased, unlike the participants in the non-exercise group. One mechanism to explain the result is the combined effect of weight loss from dietary changes in conjunction with exercise, which has been shown to effectively reduce apnea [20, 21, 22], thereby benefiting participants with OSA by potentially enhancing blood lipid profiles. Further supporting this, our mediation analysis revealed that physical activity accounts for 16.6% of the correlation between the MAP index and hyperlipidemia.

It has been reported that severe OSA significantly increased the risk of fatal cardiovascular events [23]. However, our study did not observe a statistically significant difference in the relationship between MAP index and cardiovascular mortality risk. This discrepancy may be attributed to the fact that participants at higher risk for OSA tend to prioritize preventive measures against cardiovascular events later in life as part of their daily routine. Although there was no direct association between MAP index and cardiovascular mortality, physical activity had an indirect effect in mitigating cardiovascular mortality, reducing OSA risk by 16.7%. The MAP algorithm utilized for assessing the risk of OSA severity relies on data gathered during routine primary care visits, thereby ensuring easy applicability. In the event that a patient’s screening reveals high OSA risk, the healthcare provider should consider conducting a one-night respiratory polysomnography to assess the severity of OSA and provide guidance on lifestyle modifications, such as physical exercise, to reduce cardiovascular risks. Furthermore, extensive research supports the notion that increased physical activity levels correlate with a reduced risk of CVD mortality [24]. The beneficial impact of physical exercise on cardiovascular health cannot be solely ascribed to enhancements in conventional risk factors (such as inflammatory/hemostatic biomarkers, blood pressure, lipids, and BMI [25]), but may also stem from improvements to vascular function, leading to augmented endothelium-dependent activity [26]. Given its significant benefits, moderate physical activity is vital for individuals with OSA.

Several study limitations the need to be considered when interpreting our findings. Firstly, as this was a cross-sectional study, we were unable to establish causal inferences. Secondly, as a retrospective analysis, this study is subject to potential confounding factors and inherent biases. However, leveraging the comprehensive dataset from NHANES allowed us to effectively control for potential confounding effects associated with a wide range of demographic, socioeconomic, lifestyle, and dietary factors. Thirdly, our analysis of physical activity was a qualitative, lacking detailed examination of specific aspects such as the quantity of activities. Finally, the role of continuous positive airway pressure (CPAP) therapy was not explored in this study. Therefore, future research should focus on identifying an optimal exercise program outlining the type, frequency, and intensity of exercise, as well as the duration of the program in participants at high risk for OSA. Furthermore, categorizing OSA patients into CPAP treated and untreated groups, could offer insights into how differences in physical activity levels impact the risk of cardiovascular mortality.

## 6. Conclusions

The relationship between lipid levels and the risk of OSA exhibits a non-linear pattern, a finding that remains consistent across subgroup analyses. Physical exercise indirectly influences hyperlipidemia and cardiovascular mortality by modulating MAP index. Therefore, regular physical activity may offer significant health advantages, particularly in managing OSA and its associated risks.

## Data Availability

Data are available upon reasonable request.

## Supplementary Material

Supplementary material associated with this article can be found, in the online version, at https://doi.org/10.31083/j.rcm2505175.
