# Supplementary material for: Non-Linear Association between Obstructive Sleep Apnea Risk and Lipid Profile: Data from the 2015–2018 National Health and Nutrition Examination Survey
Source: Rev Cardiovasc Med. 2024 May 17;25(5):175. doi: 10.31083/j.rcm2505175 (PMC11267181; doi:10.31083/j.rcm2505175)
Supplement: Supplementary file 1 [file 2153-8174-25-5-175-s1.docx]

# Supplemental Digital Content

**Non-linear association between obstructive sleep apnea risk and lipid profile: Data from the 2015-2018 National Health and Nutrition Examination Survey**

Contents

[Supplemental Digital Content 1](#_Toc144738402)

[Supplementary Fig. 1 Flow chart of the study. 2](#_Toc144738403)

[Supplementary Fig. 2 Predicted spline curves for the associations between the levels of lipid levels and MAP index stratified by leisure activity. 4](#_Toc144738404)

[Supplementary Table 1 Associations between MAP index score quantiles and serum lipid. 6](#_Toc144738405)

[Supplementary Table 2 Mediating effects of leisure activity on the association between MAP index and hyperlipidemia & cardiovascular mortality. 8](#_Toc144738406)

### **Supplementary Fig. 1** Flow chart of the study.


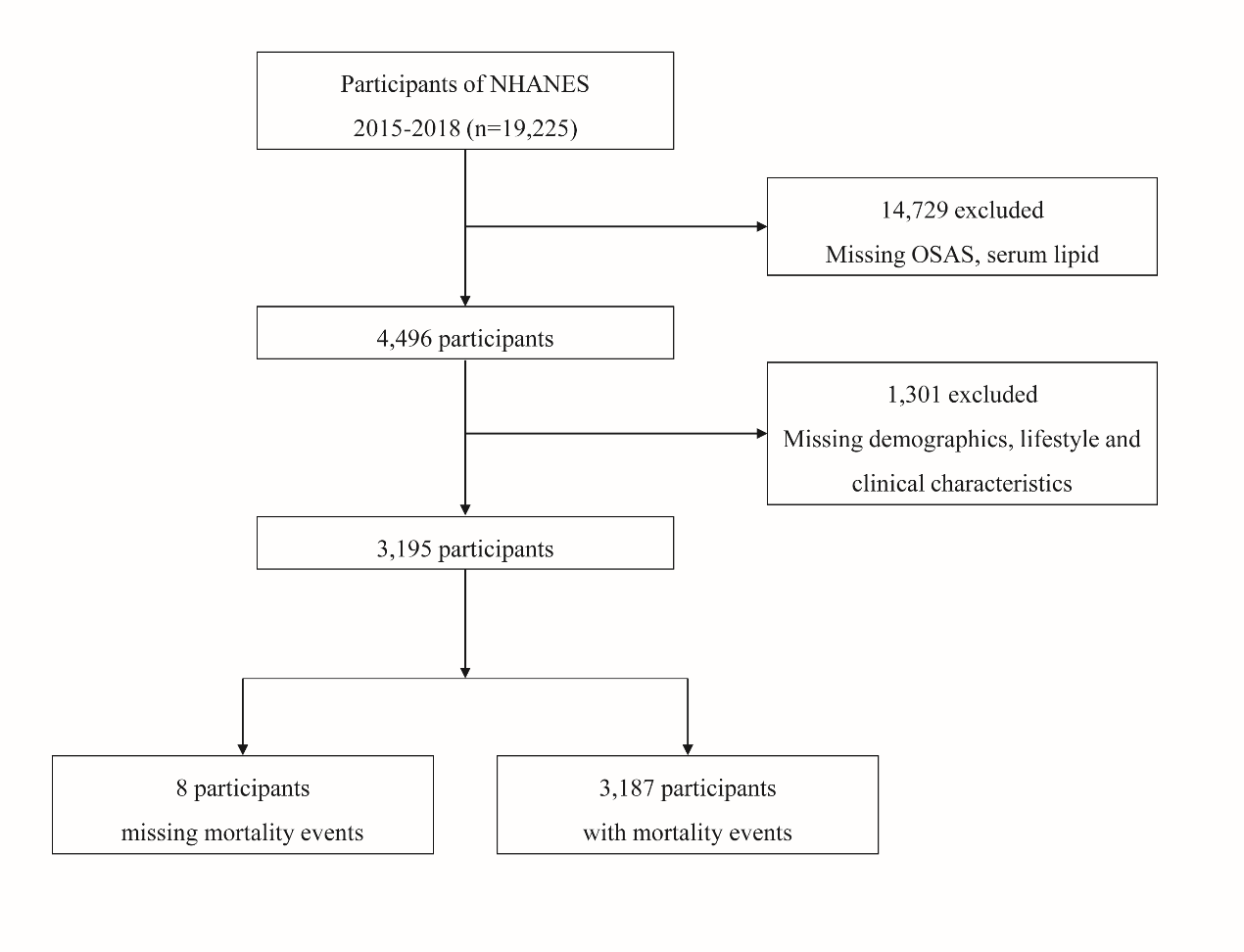


NHANES, National Health and Nutrition Examination Surveys; OSAS, Obstructive sleep apnea.

### **Supplementary Fig. 2** Predicted spline curves for the associations between the levels of lipid levels and MAP index stratified by leisure activity.


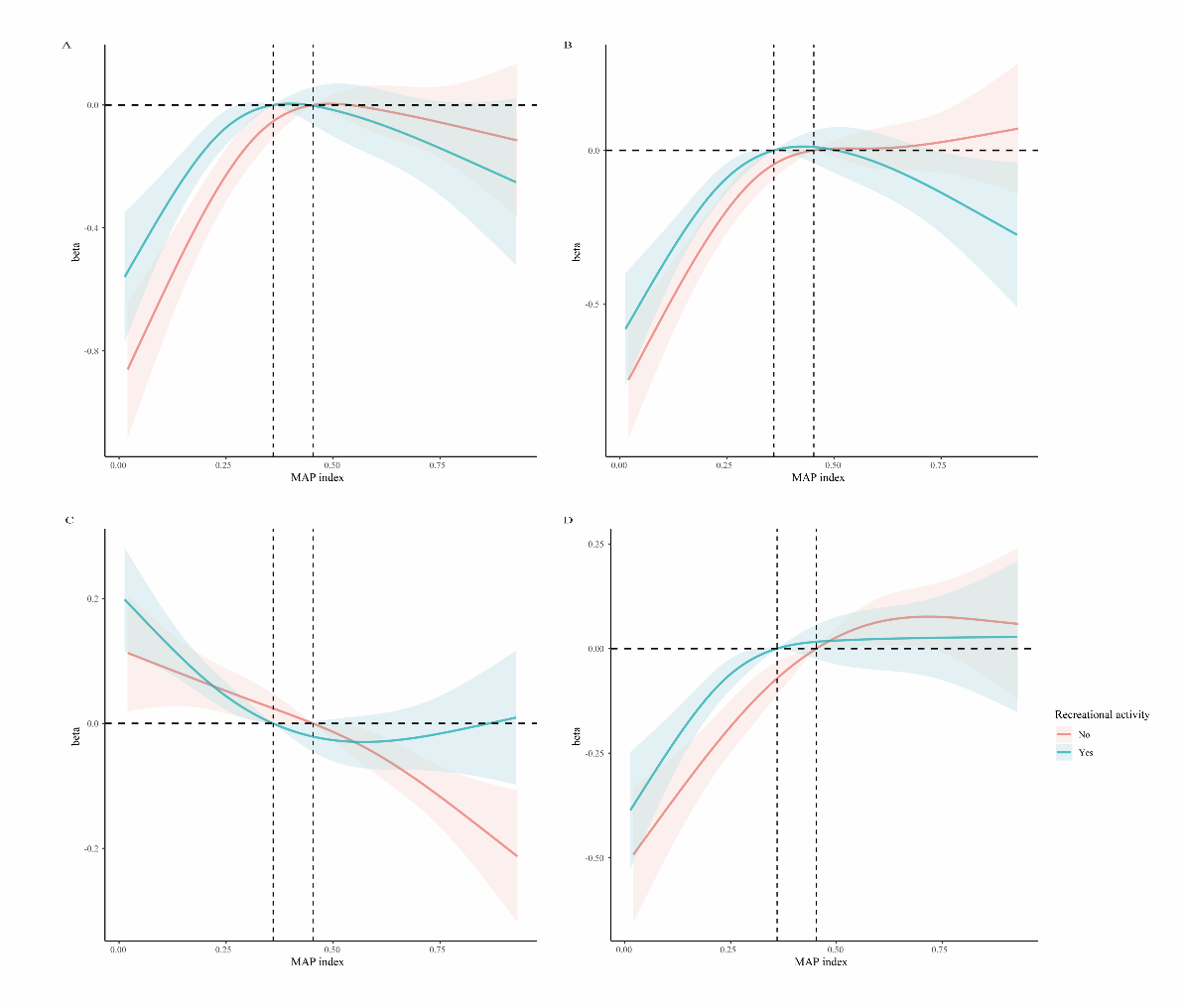


Model was adjusted for race, education, poverty, waist circumference, take anti-hyperlipidemic drug, smoking, drinking alcohol, physical activity, energy intake, and HEI. (A) TC; (B) LDL-C; (C) HDL-C; (D) TG.

### **Supplementary Table 1** Associations between MAP index score quantiles and serum lipid.

|  |  | Crude model | | Model 1 | | Model 2 | | |
| --- | --- | --- | --- | --- | --- | --- | --- | --- |
|  | MAP index | β (95% CI) | P | β (95% CI) | P | β (95% CI) | P |  |
|  | Q1 | ref |  | ref |  | ref |  |  |
|  | Q2 | 0.19 (0.06,0.32) | <0.01 | 0.26 (0.10,0.42) | <0.01 | 0.28(0.14,0.45) | <0.01 |  |
| TC | Q3 | 0.18 (0.05,0.30) | <0.01 | 0.29 (0.10,0.49) | <0.01 | 0.35(0.14,0.55) | <0.01 |  |
|  | Q4 | 0.06 (-0.11,0.22) | 0.49 | 0.24 (0.00,0.48) | 0.06 | 0.31(0.08,0.55) | 0.02 |  |
|  | P for trend |  | 0.45 |  | 0.07 |  | 0.02 |  |
|  | Q1 | ref |  | ref |  | ref |  |  |
|  | Q2 | 0.27 (0.16,0.38) | <0.01 | 0.30 (0.17,0.43) | <0.01 | 0.31 (0.18,0.47) | <0.01 |  |
| LDL-C | Q3 | 0.28 (0.17,0.39) | <0.01 | 0.35 (0.19,0.51) | <0.01 | 0.38 (0.21,0.54) | <0.01 |  |
|  | Q4 | 0.22 (0.10,0.34) | <0.01 | 0.33 (0.15,0.52) | <0.01 | 0.37(0.18,0.57) | <0.01 |  |
|  | P for trend |  | <0.01 |  | <0.01 |  | <0.01 |  |
|  | Q1 | ref |  | ref |  | ref |  |  |
|  | Q2 | -0.22 (-0.29, -0.15) | <0.01 | -0.15(-0.22, 0.07) | 0.01 | -0.13(-0.21, -0.05) | <0.01 |  |
| HDL-C | Q3 | -0.30(-0.37, -0.24) | <0.01 | -0.19(-0.28, -0.11) | <0.01 | -0.16 (-0.24, -0.08) | <0.01 |  |
|  | Q4 | -0.44 (-0.52, -0.36) | <0.01 | -0.27(-0.38, -0.16) | <0.01 | -0.23 (-0.34, -0.12) | <0.01 |  |
|  | P for trend |  | <0.01 |  | <0.01 |  | <0.01 |  |
|  | Q1 | ref |  | ref |  | ref |  |  |
|  | Q2 | 0.31(0.24,0.38) | <0.01 | 0.23 (0.13, 0.32) | <0.01 | 0.22 (0.13, 0.32) | <0.01 |  |
| TG | Q3 | 0.44(0.35,0.53) | <0.01 | 0.29 (0.17, 0.40) | <0.01 | 0.29 (0.17, 0.41) | <0.01 |  |
|  | Q4 | 0.60(0.47,0.72) | <0.01 | 0.37 (0.20, 0.53) | <0.01 | 0.37 (0.21, 0.53) | <0.01 |  |
|  | P for trend |  | <0.01 |  | <0.01 |  | <0.01 |  |

β, effect sizes.

Crude Model: no covariates were adjusted.

Model 1: adjusted for race, education, poverty, waist circumference and take anti-hyperlipidemic drug.

Model 2: race, education, poverty, waist circumference, take anti-hyperlipidemic drug, smoking, drinking alcohol, physical activity, energy intake, and HEI.

OSAS, obstructive sleep apnea; MAP, multivariable apnea prediction.

### **Supplementary Table 2** Mediating effects of leisure activity on the association between MAP index and hyperlipidemia & cardiovascular mortality.

| Outcome | Direct Effect | Indirect Effect | Proportion of indirect effect |
| --- | --- | --- | --- |
| Hyperlipidemia | 0.713 (0.603, 0.843) ^***^ | 0.920 (0.885, 0.952) ^***^ | 16.6% |
| Cardiovascular mortality | 0.705 (0.307, 1.618) | 0.916 (0.885, 0.992) ^*^ | 16.7% |

All models adjusted for race, education, smoking, and taking anti-hyperlipidemic drug,

*p < .05; **p < .01; ***p < .001
